# Assessment of Biobanking Knowledge and Attitudes towards Biospecimen Donation among Healthcare Providers in Saudi Arabia

**DOI:** 10.3390/ijerph191911872

**Published:** 2022-09-20

**Authors:** Abdelbaset Buhmeida, Mourad Assidi, Omar Alyazidi, Duaa Ibrahim Olwi, Ahmed Althuwaylimi, Fatimah M. Yahya, Leila Arfaoui, Leena Merdad, Adel Mohammad Abuzenadah

**Affiliations:** 1 Center of Excellence in Genomic Medicine Research, King Abdulaziz University, Jeddah 22254, Saudi Arabia; 2 Department of Medical Laboratory Technology, Faculty of Applied Medical Sciences, King Abdulaziz University, Jeddah 22254, Saudi Arabia; 3 Public Health Administration, Directorate of Health Affairs of Jeddah Region, Ministry of Health, Jeddah 23222, Saudi Arabia; 4 King Abdullah International Medical Research Center, Jeddah 22384, Saudi Arabia; 5 King Saud bin Abdulaziz University for Health Sciences, Jeddah 22384, Saudi Arabia; 6 MRC Epidemiology Unit, Institute of Metabolic Science, University of Cambridge, Cambridge CB2 1TN, UK; 7 School of Health and Related Research (ScHARR), University of Sheffield, Sheffield S10 2TN, UK; 8 Biochemistry Department, Faculty of Sciences, King Abdulaziz University, Jeddah 22254, Saudi Arabia; 9 Clinical Nutrition Department, Faculty of Applied Medical Sciences, King Abdulaziz University, Jeddah 22254, Saudi Arabia; 10 Faculty of Dentistry, King Abdulaziz University, Jeddah 22254, Saudi Arabia; 11 King Fahd Medical Research Center, King Abdulaziz University, Jeddah 22254, Saudi Arabia

**Keywords:** biobank, health survey, biospecimens, precision medicine, human genome project, tissue repository

## Abstract

Background: Biobanking is a critical cornerstone of the global shift towards precision medicine (PM). This transformation requires smooth and informed interaction between a range of stakeholders involved in the healthcare system. In Saudi Arabia, there is still insufficient awareness of the importance of biobanking and its potential benefits for patients, the healthcare system, and society as a whole. The purpose of this study was to determine the biobanking knowledge of Saudi healthcare providers and the potential factors that might influence their self-reported attitudes toward biospecimen donation and biobanking. Methods: A cross-sectional study was conducted targeting 636 healthcare providers in Makkah province using a structured, self-administered questionnaire. Results: The study had a response rate of 61%. The mean knowledge level about biobanks was 3.5 (±1.8) out of 7. About one-third of the participants were aware of the Human Genome Project (HGP) (35%) or the term “biobank” (34%). The mean rating of their attitude was 37.3 (±4.3) out of 55. Most participants (74%) had a positive attitude toward medical research. Job position, general health, previous blood tests, knowledge of biobanking, and attitudes toward biomedical research were significantly related and predictors of willingness to donate biospecimens (*p* < 0.05). However, concerns about biospecimen misuse and confidentiality were the main reasons for not donating biospecimens. Conclusions: This study has shown that healthcare providers mostly lack basic knowledge about HGP and biobanks and their roles and activities, and therefore are generally disinclined to actively participate in biospecimens’ collection and management. It is recommended that medical trainees receive more education and awareness about biobanks and the latest personalized healthcare approaches to improve translational research outcomes and achieve precision medicine.

## 1. Introduction

The establishment of modern biobanks, comprising huge collections of biological samples together with their biological data and research data, has been a crucial factor in the success of the Human Genome Project (HGP) [1,2]. Nowadays, high-quality and fully annotated biospecimen collections are an important prerequisite for the delivery of individualized theranostics [3,4]. Biobanks are an indispensable platform that will help shape the future of human health and address the enormous burden of diseases in countries.

To fulfill the mission of biobanks as a central platform for precision medicine (PM), there is a need for extensive collaboration, understanding, and partnership among stakeholders involved in biobanking, including the public, policymakers, patients, and medical personnel [5,6]. Although patient and public knowledge and attitudes toward tissue donation and biobanking have been reported [7,8,9,10,11], an assessment of these parameters among healthcare providers as key stakeholders in the healthcare system is needed to design more outreach and education programs. Healthcare providers are likely to be the key players in the long term, especially in biobanks that are described as hospital integrated. Research suggests that their default role as key facilitators on the patient side of biobanking has been overlooked [12]. It is primarily the physicians and their team who ask patients if they are willing to participate, provide the basic information and assurances, and manage the entire consent process.

Understanding healthcare providers’ knowledge and attitudes toward biobanking will help bridge the gap between hospitals and researchers and develop appropriate approaches for best practices [13]. It will also identify barriers to the complementarity between scientists and healthcare providers and ultimately accelerate the transition to PM [14,15]. Systematic research into healthcare providers’ knowledge and attitudes toward biobanking has just begun. An Australian study has shown that physicians strongly support the adoption of institutional biobanks. In particular, hospital-integrated biobanks require collaboration at many levels between implementation teams and healthcare professionals [15]. Another Australian study examining health professionals’ attitudes toward cancer biobanking also found that the lack of support from health professionals can hinder the appropriate implementation and routine operation of biobanks. While they were positive about government oversight of quality and compliance, it was critical to them that biobank information and researcher access be transparent [14]. In the era of OMICs, a recent study in Saudi Arabia found that there was a lack of studies that examined the attitudes of both healthcare professionals and medical students toward biobanking and biospecimen collection. Strikingly, although most Saudi medical students who participated in a previous study were in favor of the principle of biobanking, their general knowledge of the subject was quite low [16]. Positive attitudes were also observed among Malaysian healthcare stakeholders, with concerns about data and specimen protection [17]. In a study of Moroccan healthcare professionals, a similar positive attitude toward the donation of their “leftover” biospecimens was noted [18]. In a recent study of laboratory professionals in Côte d’Ivoire, investigating their knowledge of biobanks, it was found that approximately 44% of participants never heard of the term “biobank” or its existence in that country [19].

Obviously, previous studies at the local and global levels have shown that the larger healthcare stakeholders have little knowledge about the concept, function, importance, and governance of biobanks and their contribution to medical research [20,21,22,23]. Several factors such as age [22,24,25,26,27], education [1,7,8,28], and concerns about lack of privacy [29] have been shown to influence willingness to participate and/or donate biospecimens for biomedical research. In addition, religious beliefs and cultural trends have been shown to be influential, albeit insignificant, factors [17,30,31].

Since healthcare professionals are one of the key drivers of the healthcare system, they need to be provided with tailored training and continuous education to enhance their knowledge/awareness of biobanks and the new high technologies in the medical field. This is the only way to support efforts to establish a national Saudi biobank that will promote the transition toward PM [32,33]. Therefore, a better understanding of healthcare providers’ knowledge about biobanks and their attitudes toward biospecimens donation is needed before an educational program can be established. As part of the initiative to establish a nationwide biobank in Saudi Arabia, this study aimed to investigate healthcare providers’ knowledge and attitudes toward biobanking and their willingness to donate biospecimens in both government and private healthcare facilities in Makkah province, Saudi Arabia.

## 2. Methods

### 2.1. Study Design & Participants

This cross-sectional study was conducted from March 2017 to April 2018. A total of 636 healthcare providers in different governmental and private hospitals and polyclinics in Makkah province, Saudi Arabia were included in this study. All healthcare personnel, including consultants, specialists, senior house officers, health technologists, and nurses, were interviewed based on their direct involvement in healthcare services and handling of biospecimens as part of the initiative to establish a nationwide biobank facility in Saudi Arabia. The structured and self-completed questionnaires were randomly distributed and collected from consenting health professionals in different governmental and private hospitals and polyclinics in Makkah province. The researchers educated the participants about the purpose and significance of this study and explained to them that their participation was voluntary, anonymous, and confidential. Ethical approval was obtained from the Research Ethics Committee of King Abdulaziz University Hospital in Jeddah, Saudi Arabia (Ref. Number: 106-15), and special permission was obtained from each hospital/polyclinic administration to distribute the questionnaire to their employees.

### 2.2. Questionnaire

The survey was developed using a questionnaire that has been validated in previous studies [8,9,16,34]. The questionnaire underwent validation and pretesting and was improvised accordingly. It includes three sections.

(i) The first section of the survey covers the biodata including age, gender, place of residence, experience, marital status, and general health status of each participant. It recorded whether (1) they had any previous involvement with blood or organ donation, genetic testing, and/or participation in biomedical research, (2) awareness of biobanking, and (3) attitudes toward biomedical research.
(ii) The second section assessed awareness of participants’ biobanking. This section consisted of two subsections. First, the general knowledge of health care providers about biobanking was assessed and whether they were aware of the following terms: the terms “human genome project” or “biobanking”, the definition of biobanking, key terms such as consent and confidentiality, and standard operating procedures (SOPs) for biobanking. The second subsection examined the medical personnel’s attitudes toward donating biospecimens to biobanks for research purposes. Questions focused on participants’ willingness to donate tissues to biobanks in general and the possible reasons for this attitude.
(iii) Biobanks are the engine of biomedical research. Therefore, the focus of this third section was on the Research Attitudes Questionnaire, a reliable method for assessing general attitudes toward biomedical research [34]. It consists of 11 questions listed on a five-point Likert scale, with scores ranging from 1, “strongly disagree,” to 5, “strongly agree.” The total score is calculated by summing all the individual items, with higher scores indicating a more positive attitude.

### 2.3. Statistical Analysis

The measured outcomes were knowledge about biobanking and the participants’ self-expressed willingness to donate biospecimens for biomedical research purposes, as previously described elsewhere [16,34]. Willingness to donate biospecimens was categorized as a binary variable, with a score of 1 assigned for ‘yes’ and a score of 0 for ‘no’ or ‘not sure.’ For the biobanking knowledge questions, a total score was calculated by summing 7 questions, with scores ranging from 0 to 7. Each knowledge question was scored as 1 for correct answers and 0 for incorrect and ‘do not know’ answers. For the biomedical research attitude questions, a total score was calculated by adding 11 questions, with scores ranging from 11 to 55. Each attitude question was based on a five-point Likert scale, as follows: 1 ‘strongly disagree,’ 2 ‘disagree,’ 3 ‘neutral,’ 4 ‘agree,’ and 5 ‘strongly agree.’ Negative questions were reverse coded. Of note, a higher total score indicates a more positive either knowledge and/or attitude towards biomedical research.

Continuous data were presented using means and standard deviations, whereas categorical data were presented using frequencies and percentages. To determine the significant predictors of willingness to donate biospecimens, stepwise logistic regression was performed with an input of 0.05 and an output of 0.1. All tests were two-sided and a *p*-value < 0.05 was significant. The data were analyzed using STATA version 13.0 (Stata Corp, College Station, TX, USA).

## 3. Results

The study included 636 participants with a response rate of 61%. Baseline data of the study participants are shown in Table 1. The mean age was 35 ± 9.6 years, and 54% were females. The majority (70%) were or had been married. Participants were employed as consultants (6%), specialists (26%), general practitioners (13%), nurses (45%), and technologist/technician (10%). Regarding their health status, 29% reported having excellent health, and only 2% reported having fair/poor health. Thirteen percent (13%) were diagnosed with chronic diseases (hypertension, diabetes, heart disease, asthma, peptic ulcers, and cancer [35]), 27% had inherited diseases in the family, and 6% had a previous genetic test. Approximately 90% had a blood test and 42% had donated blood at least once. In addition, 12% had a tissue test and 4% had donated tissue. Less than one-third (27%) of participants were involved in medical research.

Responses to the biobanking knowledge questions are shown in Table 2. The mean score for biobanking knowledge score was 3.5 (±1.8) out of 7. About one-third of the participants were aware of the “Human Genome Project” (35%) and the term “Biobank” (34%). The majority (80%) indicated that donating a biospecimen to a biobank requires signing an informed consent form. Similarly, 61% and 69% knew that there are standard operating procedures for biospecimens’ banking and that these data must be kept confidential.

Table 3 shows participants’ attitudes toward biomedical research. The mean attitude score was 37.3 (±4.3) out of 55 points. The majority of participants (74%) had positive attitudes toward medical research. Participants expressed concern about the motivation of medical researchers. There were conflicting responses about the impact of modern science and biotechnologies. A similar pattern emerged for the statement, “A lot of emphasis on medical research and scientific progress is likely to harm research volunteers.” However, a total of 68% agreed that “medical research will find cures for many major diseases during my lifetime.”

Approximately one-third of participants (32%) were willing to donate biospecimens for medical research. Table 4 shows the associations between socio-demographic, health-related, and biobank variables and willingness to donate biospecimens. Variables that were significantly associated with a higher willingness to donate biospecimens were being a male (*p* < 0.001), consultants and specialists (*p* < 0.001), an excellent general health status (*p* = 0.014), previous blood and tissue testing (*p* < 0.001 and *p* < 0.001, respectively), previous blood and tissue donations (*p* < 0.001 and *p* = 0.018, respectively), involvement in medical research (*p* = 0.001), and a higher biobanking knowledge and a more positive attitude toward biomedical research (*p* < 0.001 and *p* < 0.001, respectively).

The logistic regression model of the significant predictors of willingness to donate biospecimens is shown in Table 5. We tested variables that were significant in the univariate analysis (gender, job position, general health, previous blood and tissue testing, previous blood and tissue donations, involvement in medical research, and biobanking knowledge and biomedical research attitude scores), in addition to age. There was significant evidence that job position was associated with willingness to donate biospecimens. Compared with technologists/technicians, nurses and general practitioners had 0.43-fold (95% CI: 0.24–0.78) and 0.37-fold (95% CI: 0.18–0.78) lower willingness to donate biospecimens, respectively. Participants with excellent health status were two times more likely to donate biospecimens than those with fair/poor health status (odds ratio [OR] = 1.88; 95% CI: 1.12–3.18). Similarly, participants who previously had a blood test (OR = 4.83; 95% CI: 1.05–22.2) were more likely to donate biospecimens than their respective counterparts. Willingness to donate increased by 25% when the biobanking knowledge score increased by one unit (OR = 1.25; 95% CI: 1.07–1.46), and a similar relationship was found when the biomedical research attitude score increased by one unit (OR = 1.20; 95% CI: 1.13, 1.28).

Reasons for willingness or reluctance to donate biospecimens are listed in Table 6. The most important factors for donating biospecimens were the promotion of medical research (84%), information about abnormal results (40%), and personal benefit (34%). On the other hand, the main factors against donating biospecimens were concerns about misuse of biospecimens in biomedical research (32%), confidentiality (27%), and possible misuse of biospecimens for commercial purposes (25%).

**Figure 1:**
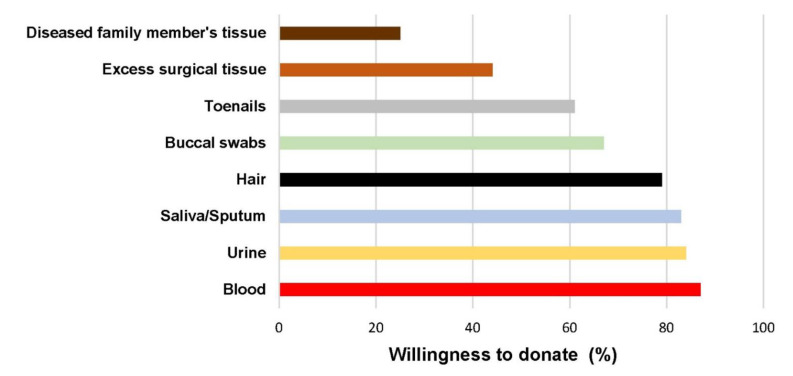
Willingness to donate a specific tissue/biospecimen.

Participants had varying degrees of willingness to donate specific tissue. Most agreed to donate blood (87%), urine (84%), and saliva/sputum (83%) (Figure 1). The willingness to donate hair (79%), buccal swabs (67%), and toenails (61%) was also widespread among medical personnel. However, participants were less willing to donate their own excess surgical tissue (44%) or the tissue of deceased family members (25%).

## 4. Discussion

Biobanking is one of the main pillars of the global transition toward precision medicine. However, there is a huge gap in the general population’s understanding, expectations, and fears about the role of biobanking in shaping the future of medical discoveries. This knowledge and attitude of the general population are critical, especially given the increasing demand for biospecimens and the enormous genomic variations and polymorphisms in the global population. The importance of successful establishment as well as effective management of biobanks and the involvement of medical staff, especially in hospital-integrated biobanks (HIBs) becomes even more important in this context. Traditional medicine relies on a reductionist approach to disease and treatment while precision medicine is a more comprehensive strategy. This “systems” approach to achieving precision medicine requires large-scale, integrated, biological data nodes [36,37,38,39]. In this context, biobanks are important platforms that store vast amounts of samples, participants’ biodata, and big data generated by high-throughput “OMICs” platforms [3,40,41,42], which contribute significantly to the implementation of precision medicine to improve the quality of healthcare services provided worldwide. Although many studies have investigated the knowledge and attitudes of the public [24,43,44,45,46,47,48], patients [43,49,50,51], and universities students’ [16,52] towards biobanks, there are few studies that focus on healthcare stakeholders, especially in developing countries such as Saudi Arabia. With this in mind, it is important to conduct an initial assessment of the general level of knowledge and attitudes toward biobanks in this geographic area before determining appropriate programs for education, awareness, and national adoption of biobanks. This is the purpose of this questionnaire-based study, which aims to determine the knowledge of the healthcare providers in Saudi Arabia about biobanks, their willingness to donate biological specimens, and the predictors of their attitudes.

This study showed that the healthcare providers who participated in this study had a lack of knowledge about biobanks. Only 34% of them were aware of biobanks and were not generally inclined to consider participating. This proportion is more in line with the findings of Lhousni et al., where 37.5% of health professionals in Eastern Morocco reported knowing about biobanks [18]. However, the knowledge of our cohort was lower than in a previous study in Côte d’Ivoire, where 56.6% of participants reported acceptable knowledge about biobanking and its role [19]. Broadly speaking, this result is to be expected since the concept of biobanking is relatively new in most developing countries, including Saudi Arabia. In addition, the participant caregivers tended not to know what biobanks are, although they did conceive them as a kind of tool involving storage and consent protocols, possibly because these are established principles in routine medical practice. They showed little understanding of the concept and function of biobanks. Moreover, most of them had never heard of the Human Genome Project.

Due to this low level of knowledge, most participants were not clear about the role and importance of biobanks. Thus, only 32% of the participants were willing to donate their biospecimens for medical research. This result was too low compared to Moroccan health professionals, 82.9% of whom showed a positive attitude towards biospecimen donation [18]. The same study reported that there was no significant association between gender and medical staff’s willingness to donate biospecimens, which is not consistent with our results. In fact, men in our cohort were almost twice as likely to donate biospecimens as female medical providers, but this attitude did not differ significantly by marital status, family status, nationality, or history of chronic diseases. Willingness to donate increased significantly with level of education and experience: 2/3 of specialists were amenable to the idea, twice as many as general practitioners, and three times as many as nurses. These findings are consistent with the study by Lhousni et al., in which they confirmed a statistically significant difference between profession and willingness to donate biospecimens. They found that physicians and senior medical staff were more willing to donate to biobanks than the others [18]. However, the results of the study by Merdad et al. conducted on senior medical students in Saudi Arabia were promising for the future, as 89% of the prospective healthcare providers expressed a positive attitude toward donating biospecimens to biobanks. Their main reasons for this willingness to support biobanks were mainly altruism and potential medical benefits for themselves or their families, as well as the promotion of medical research for future generations [16]. However, the latter results showed a lower proportion compared to Jordanian medical students, ~53% of whom had already heard about biobanks previously, and more than 90% of whom agreed or strongly agreed to participate by providing biospecimens and personal/family information [52].

Several studies have been conducted to assess the public’s knowledge and willingness to donate to a biobank in many communities that were thought to know less about biobanks compared to healthcare providers. Only about 43% of Native American participants were willing to donate to biobanks [53]. In contrast, Australians were much more willing to donate blood samples and share their personal and health data with external biobanks (>90%) [54]. Comparing the willingness of our participants to donate biological specimens to biobanks with a report published by BRO Biobank in Morocco, our target group of healthcare providers showed a higher willingness to donate various types of biospecimens. 87% and 84% of them would donate blood and urine biospecimens, respectively, compared with the 46% BRO biobank cohort. Interestingly, 44% of our participants would donate their excess surgical tissues, while 48% of Moroccan patients would donate their excess surgical tissues (including FFPE tissues and frozen tissues) to the BRO biobank [55].

People’s health status may influence their attitude and willingness to participate in biobanks through their biospecimens donation. Thus, patients’ willingness to participate in biobanks was higher when hospitalized patients and their families were approached and the procedures were simple and noninvasive [56,57]. Interestingly, patients’ families are more willing to donate to biobanks because they believe they can prevent the development of diseases in the future [57]. Abdelhafiz et al. reported that although 81% of Egyptian patients had never heard of biobanks, 85% of them showed a positive attitude towards donating their samples with their personal/health data to participate in biobanks and research activities [50,51]. This very positive attitude towards biospecimens’ donation among the Egyptian and Moroccan public (rather than medical personnel) (85% and 80%, respectively) was significantly higher than among our medical personnel (only 32%). An outgrowth of biobanks has been seen in the context of the COVID-19 pandemic, where biobanks have been significantly contributed to streamlining workflows for the rapid and safe collection of high-quality specimens and associated data. These biospecimens and associated data were rapidly shared among stakeholders globally, accelerating the understanding of the biology of the virus and thus the development of vaccines to alleviate the burden of this pandemic [58,59]. This pandemic seems to have helped promote the concept of donating biospecimens. A recent study was conducted during the COVID-19 pandemic to investigate the willingness of people to donate their remaining biospecimens from COVID-19 clinical trials for research. They reported that 80% of the participants would donate their remaining biospecimens, and 70% of them would donate their leftover/remaining specimens without signing the informed consent [60]. Therefore, it is necessary to pay more attention to biobanks to adapt and prepare them for the upcoming global health challenges/crises.

Regarding willingness to donate biospecimens, our study showed a relatively low willingness to participate among Saudi healthcare providers. These objections to biospecimen donation were generally related to concerns about misuse of the specimens and distrust of possible “commercially- oriented” biomedical research activities. In a conservative society such as Saudi Arabia, other minor reservations were related to religious and cultural reasons. These findings are consistent with previous studies that have shown that willingness to donate biospecimens is influenced by participants’ knowledge of biobanking, the type of tissue donated, the research purpose, and participants’ privacy and confidentiality [48]. These concerns are also shared by other populations such as Egyptians, Moroccans, Malaysians, and Hispanics [50,51,61]. Furthermore, some studies reported additional barriers to the willingness to donate, such as lack of publicity, fear of side effects, and being experimented on [61], age, gender, family history of diseases, previous blood or tissue donation [50], education level [50,62], and socioeconomic level [62]. In addition, more than 90% of the Australian population would donate their biospecimens to local biobanks. However, this percentage decreased to 63% were asked if they would donate their biospecimens to overseas biobanks. Furthermore, due to doubts about the commercial use of their samples, less than 59% of them would donate biospecimens to private biobanks (funded by biomedical companies), while about 94% would be willing to donate to governmental biobanks [63]. It seems that the healthcare providers participating in this study had fears of confidentiality and privacy breaches, as well as doubts about possible misuse of their personal data/biospecimens. In particular, they expressed concerns about the use of their biospecimens for research purposes that were not consistent with their values. It seems that the relatively low knowledge about biobanking procedures and their benefits are behind these fears. The lower involvement of medical caregivers in medical research is another important reason that could also justify this attitude and therefore deserves further attention.

The results of the logistic regression model showed that job position, general health status, previous blood test, biobanking knowledge scores, and attitudes toward biomedical research were significantly associated with willingness to donate biospecimens. In fact, nurses and general practitioners showed a lower willingness to donate biospecimens than others. This could be because consultants and specialists are more aware of the importance of biospecimen donation for biomedical research and therefore more willing to personally participate in a biobank. In addition, better self-reported health status was significantly associated with willingness to donate biospecimens, as reported in previous studies among the Swedish population [7] and medical students in Saudi Arabia [16]. In our study, prior blood testing was also a significant positive predictor, with 35% of participants willing to donate biospecimens compared to their respective counterparts (only 7%). Interestingly, increases in biobanking knowledge and/or attitudes toward biobanking research proved to be significant positive predictors. These findings are consistent with similar studies conducted in Italy, Sweden, and Japan, which reported that individuals who were willing to donate samples had more positive attitudes toward biomedical research than those who were not willing to donate [7,8,64]. Other studies conducted by Goddard and al. [65] and Merdad et al. [16] showed that participants who were willing to donate to biobanks had significantly higher levels of knowledge than participants who were not willing to donate.

Assuming that level of training is a predictor of willingness to support biobanks, the adoption of next-generation biobanks will necessarily require focused and vigorous training of healthcare providers—not so much in specimens’ collection and management, but in the overall rationale behind the concept of biobanking. The primary obstacle to switching to PM in Saudi Arabia appears to be mainly the lack of awareness, inadequate public education campaigns, and limited participation of health care professionals in research activities. Broad-based awareness campaigns about the role and functions of biobanks and biomedical research are needed, especially among healthcare providers and the Saudi public at large. Healthcare professionals would then be called upon by patients and policymakers to contribute more effectively to the global effort already underway to provide more efficient, participatory, and precise care, much of which relies on biobanks—a concept that seems unfamiliar at this point.

## 5. Conclusions

This study has shown a shortage of basic knowledge about HGP and biobanks and their roles and activities among healthcare providers. Therefore, they are less prone to actively participate in biospecimens’ collection and management. To overcome this unwillingness, we recommend that medical trainees receive more education and awareness about the importance of biobanks and precision medicine.

## Figures and Tables

**Table 1:** Characteristics of the study participants and biobanking-related variables (*n* = 636).

Variables	*n*	%
Age (mean ± SD)	35 ± 9.6	
Gender		
Female	338	54
Male	293	46
Marital status		
Single	187	30
Ever-married	442	70
Nationality		
Saudi Arabian	253	41
Non-Saudi Arabian	371	59
Job position		
Consultant	37	6
Specialist	154	26
General practitioner	79	13
Nurse	272	45
Technologist/technician	57	10
General health		
Excellent	185	29
Very good	244	39
Good	186	30
Fair/Poor	13	2
History of chronic disease		
No	545	87
Yes	80	13
Family history of inherited disease		
No	441	73
Yes	164	27
Previous hospitalization		
No	302	48
Yes	326	52
Previous genetic testing		
No	595	94
Yes	35	6
Previous blood testing		
No	64	10
Yes	566	90
Previous tissue testing		
No	541	88
Yes	71	12
Previous blood donation		
No	368	58
Yes	263	42
Previous tissue donation		
No	606	96
Yes	22	4
Involvement in medical research		
No	448	73
Yes	163	27

**Table 2:** Knowledge about biobanking (*n* = 636).

	*n*	%
Aware of the “Human Genome Project”	211	35
Aware of the term “Biobank”	209	34
The purpose of a biobank is to collect and store biospecimens for diagnosis, treatment, and research purposes	284	45
According to modern biobanking, biospecimens are samples and/or biomolecules with annotated clinical, socioeconomic, and lifestyle data	166	27
Donating a biospecimen to a biobank requires signing a consent form	498	80
There is a standard operating procedure for biobanks to collect, process, store, and release biospecimens	383	61
Biospecimen annotated data will be kept confidential and anonymous	428	69

**Table 3:** Attitude towards biomedical research.

	Strongly Agree *n* (%)	Agree *n* (%)	Neutral *n* (%)	Disagree *n* (%)	Strongly Disagree *n* (%)
Positive view of medical research	241 (38)	228 (36)	142 (23)	15 (2.5)	3 (0.5)
Medical researchers are mainly motivated by personal gain	94 (15)	204 (33)	204 (33)	87 (14)	35 (5)
Medical researchers can be trusted to protect by volunteering for medical research	141 (23)	263 (42.5)	192 (31)	21 (3)	3 (0.5)
Responsibility to help others by volunteering for medical research	155 (25)	255 (41)	173 (28)	35 (5)	7 (1)
Modern science does more harm than good	44 (7)	144 (23)	218 (35)	152 (25)	63 (10)
Society needs to devote more resources to medical research	142 (23)	260 (42)	191 (30.5)	24 (4)	2 (0.5)
Medical research needs to be closely regulated in order to prevent harm to research participants	200 (32)	248 (40)	155 (25)	16 (2.5)	2 (0.5)
Participating in medical research is generally safe	99 (16)	215 (34.5)	259 (42)	43 (7)	3 (0.5)
If I volunteer for medical research, I know my personal information will be kept safe and confidential	153 (24.5)	235 (38)	201 (32)	32 (5)	4 (0.5)
A lot of emphasis on medical research and scientific progress is likely to harm research volunteers	47 (7)	161 (26)	254 (41)	128 (21)	31 (5)
Medical research will find cures for many major diseases during my lifetime	130 (21)	265 (42)	193 (31)	31 (5)	6 (1)

**Table 4:** The associations between sociodemographic, health-related, and biobanking-related variables and willingness to donate biospecimens.

Variables	Not Willing to Donate	Willing to Donate	*p*-Value *
	*n* (%)	*n* (%)	
Gender			
Female	239 (74)	84 (26)	<0.001
Male	166 (60)	112 (40)	
Marital status			
Single	118 (67)	57 (33)	0.995
Ever-married	286 (67)	138 (33)	
Nationality			
Saudi	150 (64)	83 (36)	0.244
Non-Saudi	249 (69)	112 (31)	
Job position			
Consultant	14 (40)	21 (60)	<0.001
Specialist	80 (54)	67 (46)	
General practitioner	55 (71)	22 (29)	
Nurse	205 (79)	53 (21)	
Technologist/technician	34 (64)	19 (36)	
General health			
Excellent	106 (60)	70 (40)	0.014
Very good	167 (72)	65 (28)	
Good	127 (72)	50 (28)	
Fair/Poor	6 (46)	7 (54)	
History of chronic disease			
No	358 (69)	162 (31)	0.111
Yes	46 (60)	31 (40)	
Family history of inherited disease			
No	277 (66)	140 (34)	0.698
Yes	109 (68)	51 (32)	
Previous hospitalization			
No	205 (69)	90 (31)	0.377
Yes	201 (66)	103 (34)	
Previous genetic testing			
No	392 (68)	183 (32)	0.127
Yes	14 (54)	12 (46)	
Previous blood testing			
No	56 (93)	4 (7)	<0.001
Yes	353 (65)	188 (35)	
Previous tissue testing			
No	358 (70)	155 (30)	<0.001
Yes	34 (49)	36 (51)	
Previous blood donation			
No	265 (74)	93 (26)	<0.001
Yes	143 (59)	101 (41)	
Previous tissue donation			
No	400 (68)	184 (32)	0.018
Yes	7 (41)	10 (59)	
Involvement in medical research			
No	309 (72)	122 (28)	0.001
Yes	86 (57)	66 (43)	
Age (mean ± SD)	34.7 ± 9.5	36.1 ± 10.1	0.095
Biobanking knowledge score (mean ± SD)	3.2 ± 1.8	4.3 ± 1.5	<0.001
Biomedical research attitude score (mean ± SD)	36.0 ± 3.3	39.7 ± 4.7	<0.001

* Chi-square test was used except for age, biobanking knowledge score, and biomedical research attitude score where a *t*-test was used.

**Table 5:** Logistic regression model of the significant predictors of willingness to donate biospecimens.

Variables	OR	SE	95% CI	*p*-Value
Job position (technologist/technician = reference)				
Nurse	0.43	0.13	(0.24, 0.78)	0.004
General practitioner	0.37	0.14	(0.18, 0.78)	0.009
General health (fair/poor = reference)				
Excellent	1.88	0.50	(1.12, 3.18)	0.017
Previous blood testing (no = reference)				
Yes	4.83	3.76	(1.05, 22.2)	0.043
Biobanking knowledge score	1.25	0.10	(1.07, 1.46)	0.005
Biomedical research attitude score	1.20	0.04	(1.13, 1.28)	<0.011

Pseudo R^2^ = 0.23. *Abbreviations:* OR, odds ratio; SE, standard error; CI, confidence interval.

**Table 6:** Reasons for willingness or unwillingness to donate.

Variables	*n*	%
*Reasons for willing to donate (n = 196)*		
The biobank will advance medical research and benefit society and future generations	153	84
My family and I will benefit	62	34
I could be notified about abnormal results	72	40
Samples will already be collected as part of my medical care	40	22
Biobanks are already established as the core facility of biomedical research in developed countries	51	28
*Reasons for not willing to donate (n = 410)*		
Concern about the misuse of biospecimen in biomedical research	106	32
Concern about discovering genetic predispositions to some diseases	70	21
Concern about confidentiality	89	27
Concern that genetic information may be used for discriminatory purposes	51	15
Concern that biospecimen may be used for commercial purposes	83	25
Fear of needles/injections	73	22
Religious reasons	56	17

## Data Availability

Raw data is available upon reasonable request.

## Abbreviations

GPA: Grade-point average; HGP: Human genome project; KAU: King Abdulaziz University; PM: Precision medicine; RAQ: Research attitudes questionnaire; SOPs: Standard operating procedures.
